# Paralytic Shellfish Poisoning (PSP) Toxins in Bivalve Molluscs from Southern Italy Analysed by Liquid Chromatography Coupled with High-Resolution Mass Spectrometry (UHPLC-HRMS/MS)

**DOI:** 10.3390/toxins16110502

**Published:** 2024-11-20

**Authors:** Pasquale Gallo, Sara Lambiase, Ida Duro, Mauro Esposito, Angela Pepe

**Affiliations:** 1 Istituto Zooprofilattico del Mezzogiorno, Via Salute 2, Portici, 80055 Naples, Italy; pasquale.gallo@izsmportici.it (P.G.); ida.duro@ats-brescia.it (I.D.); mauro.esposito@izsmportici.it (M.E.); angela.pepe@izsmportici.it (A.P.); 2 ATS di Brescia, Viale Duca degli Abruzzi, 25124 Brescia, Italy

**Keywords:** algal biotoxins, accurate mass spectrometry, PSP toxins, Q-Exactive mass spectrometer, molluscs

## Abstract

A new method for simultaneous determination by liquid chromatography coupled with high resolution mass spectrometry (UHPLC-HRMS/MS) of 14 paralytic shellfish poisoning toxins (PSP), that is, Saxitoxin, Neosaxitoxin, Gonyautoxins and their respective variants, in bivalve molluscs, is herein described. The samples were extracted by acetic acid solution, then analysed by UHPLC coupled with a Q-Exactive Orbitrap Plus high resolution mass spectrometer, by electrospray ionization mode (ESI) with no further clean up step. The analysis was carried out by monitoring both the exact mass of the molecular precursor ion of each compound (in mass scan mode, resolution at 70,000 FWHM) and its respective fragmentation patterns (two product ions) with mass accuracy greater than 5 ppm. The analytical performance of the method was evaluated calculating trueness, as mean recoveries of each biotoxin, between 77.8% and 111.9%, a within-laboratory reproducibility (RSD_R_) between 3.6% and 12.2%, the specificity, the linearity of detector response, and the ruggedness for slight changes The results of the validation study demonstrate this method fits for the purposes of the official control of PSP toxins in molluscs. The results of two years of monitoring in local mussel farms are also reported, showing that no significant concerns for food safety in the monitored productions.

## 1. Introduction

Saxitoxin and 57 related compounds are a group of neurotoxins produced by microorganisms living in marine environment [1]. Saxitoxin (STX) is the most potent of these biotoxins, that can cause an acute human neurological syndrome, known as paralytic shellfish poisoning (PSP); for this reason, they are also referred to as PSP toxins [2,3,4].

Saxitoxin and the other PSP toxins are produced by several species of dinoflagellates such as *Alexandrium* (*A. tamarense*, *A. catenella*, *A. fundyense*, *A. minutum*, *A. cohorticula*, *A. angustitabulatum*, *A. ostenfeldii*, etc.), *Gymnodinium catenatum* and *Pyrodinium bahamense* (var. *P. bahamense compressum*) in marine environments [3]. These neurotoxins can accumulate in high concentrations in filter-feeding organisms, mainly bivalve molluscs, as well as in plankton eaten by some species of fish and crustaceans, hence they can also accumulate in several predator fish species [2,4,5]. Through this pathway, PSP toxins can enter into the food chain, representing a serious threat to human health. In fact, while PSP toxins do not cause any damage to shellfish, but in humans the consumption of contaminated food can cause a serious foodborne disease. PSP toxins block reversibly voltage-gated sodium channels of muscle and nervous tissues resulting in harmful gastrointestinal, neurological and cardiovascular effects, including headache, quadriplegia, excitement, vomiting, nausea, and diarrhoea, up to provoking respiratory paralysis in the worst cases [3,6,7].

PSP toxins have a tetra-hydropurine molecular structure, showing variable hydrophilic or hydrophobic substituent side chains. The most common and studied PSP toxins are those showing the hydrophobic substituent. They belong to different sub-groups, as follows: the carbamoyl compounds (Saxitoxin, Neosaxitoxin and Gonyautoxins 1-4) and their respective decarbamoyl-derivatives (dc-Saxitoxin, dc-Neosaxitoxin and dc-Gonyautoxins 1-4); the N-sulpho-carbamoyl toxins (Gonyautoxins 5-6, Gonyautoxin C1-4); and hydroxylated saxitoxins [1,8].

PSP toxins show different toxicity that depends on the structure of their side chains, which affect the binding affinity to the sodium channels in humans. The most toxic are the carbamoyl-PSPs, even though STX is recognised as the most harmful compound; hence, the toxicity of the other related toxins is expressed in comparison with STX by toxicity equivalent factors (TEFs) [5,8,9].

Due to their high toxicity to humans, sensitive and reliable test methods are necessary to monitor the presence of PSP neurotoxins in seafood, to protect consumers’ health and avoid economic losses for mollusc breeders. Firstly, a biological test was introduced to detect PSP toxins in seafood by the so-called mouse bioassay (AOAC n. 959.08) [10]. Later, chemical methods based on high performance liquid chromatography and fluorescence detection were developed, introducing a pre-column oxidation of toxins (AOAC n. 2005.06) [11], and subsequently a post-column oxidation (AOAC n. 2011.02) [12], to increase analysis sensitivity, reliability and identification of the single PSP toxins. The mouse bioassay (MBA) was recognised as the reference method by the Commission Regulation (EC) n. 2074/2005 until 31 December 2018 [13], although it was a controversial procedure, because of concerns, and, above all, low sensitivity, poor reproducibility and some matrix interference.

Thus, since the Commission Regulation (EU) n. 2017/1980 [14] entered into force, the MBA was replaced by the so-called Lawrence method, based on a high-performance liquid chromatography (HPLC) analysis with pre-column oxidation and fluorescence detection (AOAC n. 2005.06) [11]. This currently represents the reference method for both monitoring official control activity and for controversy action regarding the results. The AOAC Method n. 2005.06 [11] is a highly sensitive and specific method for the analysis of PSP toxins, but it is a complex and time-consuming procedure, as it involves many analytical steps, including derivatization of the analytes by periodate, then peroxide oxidation. Moreover, the oxidation of different toxins may lead to the same reaction products, making their identification and quantification very difficult [8].

As a result, in recent years, methods based on liquid chromatography coupled to low resolution tandem mass spectrometry detection on triple quadrupole (LC-MS/MS) have been developed to analyse PSP toxins [3,5,6,15,16]. LC-MS/MS on triple quadrupole is a highly sensitive and selective technique, which provides reproducible and accurate results, and allows to simplify sample preparation avoiding oxidation steps. Anyway, all these methods introduced a clean up step by SPE cartridge. Also, liquid chromatography coupled to high resolution Fourier transform mass spectrometry on Orbitrap (LC-HRMS) has been employed, to increase selectivity of the method by monitoring the accurate mass of each PSP toxin [17], but even in that work a clean up purification step was necessary.

In this study, to overcome some limits of the Lawrence method (AOAC n. 2005.06) [11], we describe a new procedure for the determination of 14 PSP toxins in fresh and canned bivalve molluscs, by liquid chromatography coupled to high resolution tandem mass spectrometry (UHPLC-HRMS/MS) on a Q-Exactive Orbitrap Plus. In our method, the optimisation of chromatography and mass spectrometry detection allowed us to simplify and speed up sample preparation, reduce analysis time, and improve sample throughput, as no sample clean up by SPE is required.

In particular, we developed a quantitative method for simultaneous identification and quantification of 14 PSP toxins, including Saxitoxin (STX), Decarbamoyl-saxitoxin (dc-STX), Gonyautoxin 1 (GTX 1), Gonyautoxin 2 (GTX 2), Gonyautoxin 3 (GTX 3), Gonyautoxin 4 (GTX 4), Gonyautoxin 5 (GTX 5), Gonyautoxin 6 (GTX 6), Neosaxitoxin (NEO), Decarbamoyl-neosaxitoxin (dc-NEO), Decarbamoyl-gonyautoxin 2 (dc-GTX 2), Decarbamoyl-gonyautoxin 3 (dc-GTX 3), N-Sulfocarbamoyl-gonyautoxin 1 (C 1) and N-Sulfocarbamoyl-gonyautoxin 2 (C 2). The method was validated in-house, then accredited by the Italian Accreditation Body, according to the UNI EN ISO/IEC 17025:2018 international standard, used for the official control of mussel farms within local monitoring plans. The method we developed fulfils the recommendation by the Italian Reference Laboratory (NRL-BM, Centro Ricerche Marine, Cesenatico, FC), to introduce new and better performing test procedures, alternative to the Lawrence method. Actually, the UHPLC-HRMS/MS method described here is the only one accredited in Italy for the determination of 14 PSP toxins in molluscs.

## 2. Results and Discussion

### 2.1. Optimisation of Sample Preparation

For sample preparation, the liquid extraction step was carried out using a solution of aqueous acetic acid, similar to the procedure described by the Lawrence method (AOAC, n. 2005.06) [11] and also adopted also by Ochi [6]. The use of aqueous acetic acid as an extracting solvent instead of hydrochloric acid, employed in the MBA test, allows us to obtain the real profiles of these neurotoxins in the samples. In fact, it is known that HCl extraction leads to the conversion of some PSP toxins into their more toxic analogues, thus resulting in an overestimation of the total equivalent toxicity calculated. The rate of conversion depends on the pH of the solution, and it is reduced when the pH is adjusted to around 3. Experimental evidence shows that aqueous acetic acid extraction prevents this conversion [8]. Moreover, the extraction efficiency using aqueous acetic acid was comparable to the extraction by mixtures of organic solvents, such as acetonitrile/water 80/20 containing 0.1% formic acid [3,18].

An important issue concerning the development of an analytical LC-MS method is represented by the matrix effect, that can usually reduce the desolvation and/or ionization of target molecules, thus decreasing test sensitivity and precision. As a matter of fact, molluscs contain proteins, aminoacids and minerals that are co-extracted with PSP toxins, interfering with their determination [6,19,20]. For these reasons, sample clean up, usually by SPE cartridges, is often necessary to improve analytical results. The method we developed does not require a purification step after extraction, but only a solvent dilution step by the LC mobile phase, followed by analysis by UHPLC-HRMS/MS on the Q-Exactive Orbitrap Plus. Instead, the Lawrence reference method requires two different SPE purification steps for complete analysis of all PSP toxins. In our experiments we observed that a 1 to 5 extract dilution by the LC mobile phase reduces the effects of matrix interference by ions from salts and proteins. The results, in terms of mean recoveries and precision, were better than those obtained by analysing the same spiked samples using the Lawrence reference method.

### 2.2. Optimisation of Chromatography

**Figure 1:**
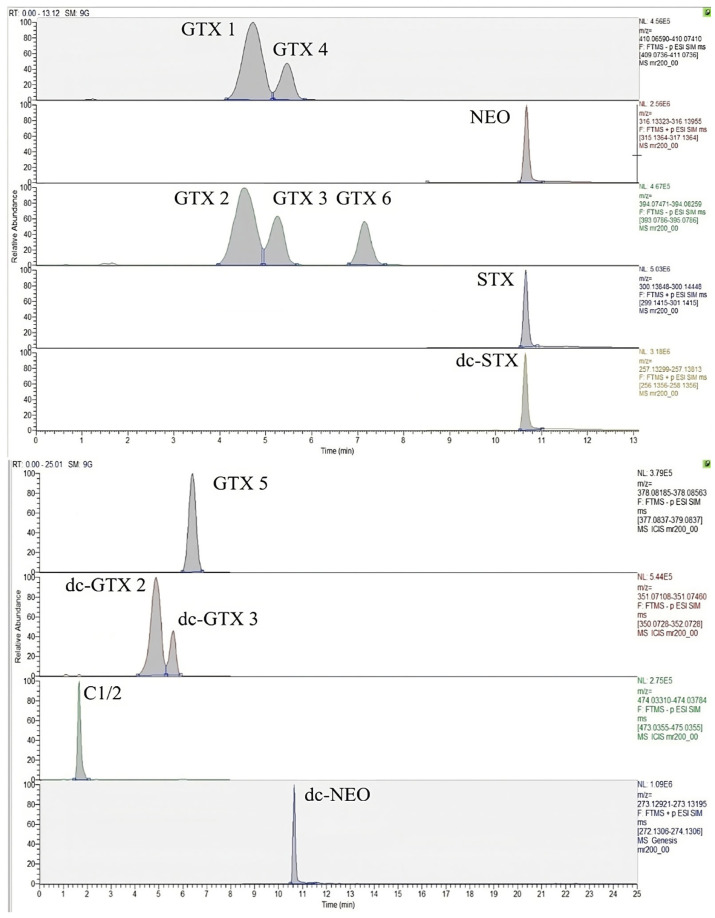
UHPLC-HRMS tSIM chromatograms, in negative and positive ion mode, of the 14 PSP toxins studied in a matrix-matched standard solution at 25–211 ng mL^−1^.

HILIC is suitable for the separation of molecules with polar moieties, like PSP toxins. For the development of this method, the TSK gel Amide 80 column chosen retains effectively the pyrrolopurine-base structure of the PSP toxins; the same column was recommended by other authors [18]. Moreover, this column allowed us to obtain the best results in terms of analyte separation, although it was reported that it does not completely separate some toxins [6]. In fact, under the optimized chromatographic conditions reported below, the TSK gel Amide 80 column was able to separate completely all the toxins, including the most critical epimeric pairs, with the exception of the epimers C 1 and C 2. In Figure 1 the tSIM chromatograms of accurate mass precursor ions of a matrix matched standard mix of 14 PSP toxins at 25–211 ng mL^−1^ are reported.

**Figure 2:**
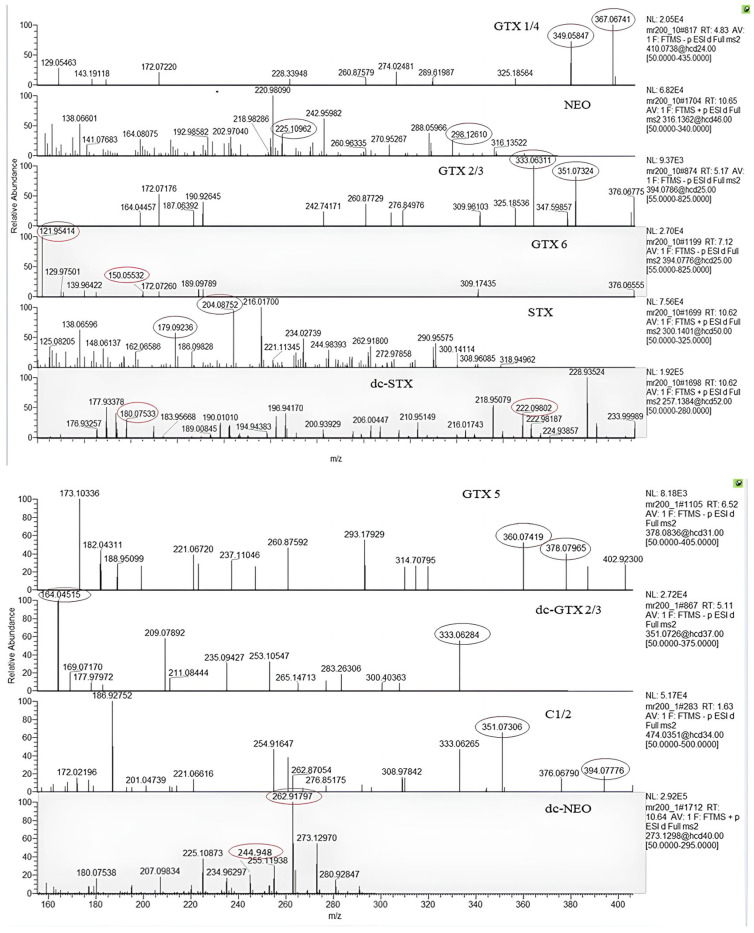
The ddMS2 spectra of the 14 PSP toxins studied in a matrix-matched standard solution at 25–211 ng mL^−1^. The accurate mass of the product ions of each PSP toxin is shown in the circles.

As described by Ochi (2021) [6], working in low resolution mass spectrometry by triple quadrupole, the main problem concerns the identification of the toxins C 1, C 2, GTX 2, GTX 3 and GTX 6 because they share the same ion transitions at *m*/*z* 396 → 316 amu and *m*/*z* 396 → 298 amu, that are monitored for these compounds. We improved the chromatographic separation of all toxins, apart from C 1 and C 2; it is noteworthy the analysis in high resolution mass spectrometry allowed us to overcome the problem derived by monitoring compounds with the same ion transitions. In Table 1, the exact mass of the precursor ions monitored are reported; based on the accurate mass selection of precursor ions, the ddMS2 chromatograms of a matrix matched standard mix of 14 PSP toxins at 25–211 ng mL^−1^ account for high specificity of the method (Figure 2). In this figure the chromatograms of the quantifier ion for each PSP and their corresponding MS/MS spectra are reported.

Therefore, for these PSP toxins, the complete separation of the chromatographic peaks is necessary for their correct identification and quantification. The main challenge was the separation of the epimers C 1 and C 2 because these two toxins are eluted early, therefore the chromatographic optimisation for these two compounds was more difficult. For this reason, in order to obtain the most accurate quantification, we preferred to calculate the C 1 and C 2 amount as their sum (C 1/2 sum). It was considered that this approach did not affect the performance of the method because the contributions of C 1 and C 2 to the total STX equivalent toxicity are lower than other PSP toxins, considering the TEFs proposed by the EFSA [8].

The retention time reproducibility was fundamental in the optimisation of the chromatographic method, since it affects analyte identification. In fact, a significant shift of the retention times, due to the matrix effect, was observed for spiked samples if compared to the standard working solutions. The identification of the toxins proved to be complex, as previously described by other authors [18]. To avoid shifts in retention times and improve quantitative results, the matrix-matched calibration curve approach was introduced; moreover, the best results in terms of retention time reproducibility were obtained when both standard working solutions and samples were diluted 1 to 5 by the LC mobile phase, because the interference from the matrix are minimised.

The column temperature is critical for both separation and peak shapes in the chromatographic analysis; in our method, we observed that the optimal temperature for separation and peak sharpness in the TSK gel Amide 80 column is at 30 °C.

### 2.3. Optimisation of Mass Spectrometry Determination

The relative intensities of all the precursor and product ions were optimised to improve signal to noise ratios. The analysis in both positive and negative ionization mode allows for simultaneous determination of each PSP toxin. The selected products ions were monitored in tSIM-ddMS2 scan mode for unambiguous identification of all toxins. The precursor and product ions of each PSP toxin are summarised in Table 1. For quantification, the precursor ion of each toxin was monitored, and the concentrations in samples were calculated by interpolation of the linear regression of matrix-matched calibration curves; the identification based on the presence of corresponding MS/MS diagnostic product ions allows for highly selective and sensitive determination.

### 2.4. Method Validation

Method validation was performed according to the European Union requirements [21] and the EURACHEM guidelines “The Fitness for Purpose of Analytical Methods” [22]. The analytical performance of the method should be proved to ensure its reliability and ruggedness for the aims of official controls. Thus, method selectivity, trueness, precision in terms of repeatability and within-laboratory reproducibility, the linearity of detector response, account for adequate analytical performance along time. The limits of quantification (LOQs) indicate if the method is adequate for purposes in respect to the maximum tolerable limits set by the law.

Selectivity is an important parameter to exclude the presence of compounds from matrix interfering with the analytes of interest; it was assessed by the absence of signals in the ddMS2 spectra of each PSP toxin, close to their retention times. The measurements of accurate mass of both the precursor and two product ions, highly improve sensitivity by increasing the signal to noise ratio. Specificity is evaluated by the absence of substances interfering with detection and the unambiguous confirmation of the PSP toxins in their tSIM-ddMS2 chromatograms; the validation study showed no chromatographic interference with the target PSP toxins.

The linearity of the detector response was tested by both solvent and matrix-matched calibration curves (Table 2). Their comparison showed a significant matrix effects for all PSP toxins. The coefficients of bivariate correlation, r^2^, were all higher than 0.99 in concentration ranges spanning over two orders of magnitude, accounting for very good linearity of the high resolution mass spectrometer Orbitrap, in concentration ranges adequate for the official control of seafood.

Regarding the LOQs of the method, considering that we introduced a novel HRMS/MS detection to be compared to the HPLC-FLD Lawrence procedure, we referred to the same values reported in the method AOAC n. 2005.06 [11] and the standard UNI EN 14526: 2017 [23]. These LOQs were experimentally verified and confirmed by our method, proving the UHPLC-HRMS/MS procedure is at least equivalent to the reference method for determination of all the PSP toxins but at the same time provides unambiguous identification.

The data regarding method trueness and precision are reported in Table 3. Trueness was calculated for all the PSP toxins as the mean percentage recoveries, at two spiking levels. No analytical performance criteria has been set by the European Union legislation about methods for determination of biotoxins. Starting from the concentration ranges of PSP toxins usually detectable in molluscs, we considered acceptable mean recoveries between 70 and 120%. As it can be seen, the mean recoveries are within this acceptability range for all the biotoxins, accounting for good or excellent method trueness, thanks to the accurate mass measurements and the use of matrix-matched calibration curves that increased method sensitivity and specificity.

Method precision was evaluated at two spiking levels: through repeatability, that is, the variance of replicate tests from a single analyst in the same working batch, and within-laboratory reproducibility, by the variance of the measurements from two or more analysts during different working sessions. The relative standard deviations calculated for repeatability and within-laboratory reproducibility, respectively RSD_r_ and RSD_R_, respectively, account for method variability over time. Unlike trueness, repeatability and reproducibility values < 15% are required by both the Lawrence reference method AOAC n. 2005.06 [11] and the UNI EN 14526: 2017 international standard [23]. The results show that the method has excellent repeatability and within-laboratory reproducibility, well below the reference value, for all toxins and regardless of the contamination levels.

The validation study demonstrated that the method has LOQs from 97 µg kg^−1^ for the sum of C1/2 down to 9 µg kg^−1^ for dc-STX and dc-GTX, and good-to-excellent trueness and precision data for all toxins. Moreover, the high selectivity and specificity, as well as the linearity of detector response, make the method effective for the official control of PSP toxins contamination in seafood.

### 2.5. Quality Assurance of Performance by Proficiency Tests

The laboratory for official control of food must participate in proficiency test programmes (PTs) for quality assurance of results. For the PSP toxins in molluscs we participate to proficiency tests provided by the Organisation of Interlaboratory Studies WEPAL-QUASIMEME and by the Italian Reference Centre for monitoring of marine biotoxins (NRL-BM). We applied the UHPLC-HRMS/MS method since 2021 for PT sample analysis. The results were always satisfactory, being our z-scores in the range ±2. As an example of method performance, the results regarding natural contaminated samples provided in a proficiency test by the NRL-BM are reported and discussed. The samples were analysed by both the UHPLC-HRMS/MS method and the HPLC-FLD Lawrence reference AOAC n. 2005.06 test, to compare performance (Table 4). The measurement uncertainty is calculated according to the Horwitz equation.

The PT aimed to identify and the quantify individual PSP toxins, calculating their concentration both as individual toxins and as total STX equivalent toxicity. The toxins dc-STX, GTX 6, NEO, dc-NEO, dc-GTX 2, dc-GTX 3 were not detected at all. The results calculated using both methods were satisfactory, and comparable for STX, GTX 2/3 sum, GTX 5, C 1/2 sum, STX equivalents. On the other hand, the UHPLC-HRMS/MS method allows to separate and determine GTX 1 and GTX 4, thus their sum is not comparable. It is noteworthy the values of total STX equivalent toxicity calculated by both methods are very close.

These results prove that the UHPLC-HRMS/MS method is reliable and reproducible. The measurements by high-resolution accurate mass spectrometry on the hybrid quadrupole-Orbitrap Q-Exactive Plus mass spectrometer ensure higher selectivity and sensitivity with respect to the Lawrence method by HPLC with florescence detection. Unambiguous identification of PSP toxins by high resolution MS/MS spectra as well as increased sensitivity and specificity of the method are remarkable features for official control analysis.

### 2.6. Monitoring Data

The monitoring of all the mussel farms in the Campania region for the official control of possible health risks derived from PSP toxins was successfully carried out in 2022 (204 samples), 2023 (189 samples) and 2024 (first six months, 94 samples). All the samples were mussels of the species *Mytilus galloprovincialis*, collected evenly during all the months of the year. This product represents a relevant economic resource in the Region, moreover is very popular as food, thus a constant monitoring activity is carried out by the Local Health Authorities, to ensure consumer’s health. Our novel method allows for rapid and reliable control activity to support Local Health Authorities. PSP toxins were not detected at all in the 487 samples analysed during this monitoring period, accounting for the very good quality of our mussel productions. In the period monitored no algal bloom was observed along the coasts of the Region.

## 3. Conclusions

The contamination by PSP toxins is a health risk for consumers, and requires attention for the safe management of mollusc productions. Italy is surrounded by different marine environments (Northern and Southern Tyrrhenian Sea, Adriatic, Ionian Sea); showing wide condition variability, moreover, climate change has been affecting these environments for several years, causing increasing algae contamination and consequently possible marine biotoxin outbreaks. For these reasons, the Italian Health Authorities issued regional monitoring plans and provided action levels for contamination by marine biotoxins, to protect both consumer health and mollusc productions. It is likely that the increasing import of seafood from countries outside of the EU (bordering the southern Pacific Ocean or the Atlantic Ocean) requires more control activity for possible contamination by marine biotoxins. In this scenario, it is relevant to carry out reliable and rapid official control analysis of molluscs.

In this work, we describe a new UHPLC-HRMS/MS method for the quantification and unambiguous identification of 14 PSP toxins on the basis of accurate mass measurements by the Q-Exactive Orbitrap Plus mass spectrometer. The method was developed by simplifying sample clean up, which was performed only by liquid extraction, with no further purification step, like the SPE chromatography required by the Lawrence reference method n. AOAC 2005.06. The introduction of sample dilution and the matrix-matched calibration curve allows for direct UHPLC-HRMS/MS determination, increasing sensitivity and specificity thanks to accurate mass monitoring. As a result, the method allows us to determine PSP toxins down to low concentration levels, reducing time and cost analysis. Higher sample throughput and mass spectrometry determination make it possible to perform more analyses quickly, thus allowing for prompt follow up actions in the case of non-compliant contamination by PSP toxins. This is more advantageous than the Lawrence reference method, based on liquid chromatography coupled with a fluorescence detector, which requires several working steps for the analysis of all the PSP toxins. In our opinions, the UHPLC-HRMS/MS method by accurate mass measurements using the Orbitrap exhibits analytical performance better than the Lawrence reference method, as observed by their comparison during proficiency tests. Moreover, the Italian National Reference Center encourages the introduction of MS-based methods to improve control activity. For these reasons, the UHPLC-HRMS/MS method using the Orbitrap is adequate for a modern and more effective official control of PSP toxins contamination and is successfully applied for monitoring plans regarding mussel production and imported commodities.

## 4. Materials and Methods

### 4.1. Mollusc Samples

For method development and validation, some samples of bivalve molluscs, including mussels, oysters, clams and scallops, were used as blank materials. The samples were previously collected during the official control activities. The molluscs, after sampling, were transferred to our laboratory and stored frozen at −20 °C until analysis.

### 4.2. Reagents and Chemicals

The standard stock solutions of the PSP toxins, that is, STX, NEO, dc-STX, dc-NEO, a mix of GTX 1 and GTX 4 (combined), a mix of GTX 2 and GTX 3 (combined), GTX 5, GTX 6, a mix of C 1 and C 2 (combined) and a mix of dc-GTX 2 and dc-GTX 3 (combined), were purchased from the Institute for Marine Biosciences, National Research Council of Canada (NRC, Halifax, Nova Scotia, Canada); all of the stock solutions were in aqueous solution, adjusted at pH between 3.4 and 6 with acetic acid or hydrochloric acid (Table S1). For method validation and sample analysis, a standard mix solution containing all of the PSP toxins was prepared as described in Table S1; exact volumes of each toxin were collected and added to 305 µL of HPLC grade water to obtain the final volume at 1000 µL (Table S1). Working standard solutions were prepared by dilution of the standard mix solution, as described in Table S2. High purity solvents, suitable for HPLC and mass spectrometry analysis, were used. Acetonitrile (ACN) and formic acid (HCOOH) were purchased from Carlo Erba, glacial acetic acid (CH_3_COOH) from Sigma Aldrich (Darmstadt, Germany) and ammonium formate (NH_4_HCO_2_) from VWR (Radnor, Pennsylvania, USA). HPLC grade water was produced in-house by a Millipore system (Millipore, Billerica, MA, USA).

### 4.3. Sample Preparation

**Figure 3:**
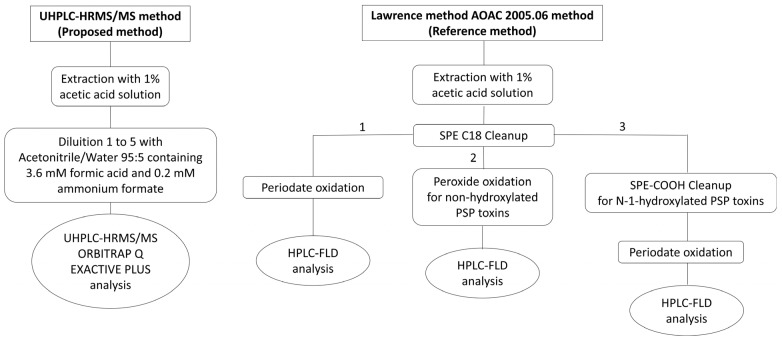
Flow diagram comparing the UHPLC-HRMS/MS method and the reference Lawrence method n. AOAC 2005.06. The SPE-COOH cleanup (3) is used only for extracts containing N-1-hydroxylated PSP toxins.

The shells of the mussels were removed, and then the sample was grinded; for sample extraction, 5.00 ± 0.01 g of homogenised tissue was mixed with 3 mL of 1% acetic acid solution and placed into a water bath at 100 °C for 5 min. After mixing by vortex, cooling and centrifugation (2246× *g*, 10 min, 4 °C), the supernatant was transferred to a 10 mL graduated cylinder and the extraction process was repeated, once again adding 3 mL of 1% acetic acid solution. The supernatants were combined and brought to the final volume of 10 mL with HPLC grade water. Then, 300 µL of the final extract was diluted with 1200 µL of acetonitrile/water 95/5 *v*/*v*, containing 3.6 mM formic acid and 0.2 mM ammonium formate (mobile phase B) and injected into the chromatographic system; a comparing this method with the Lawrence method is shown (Figure 3).

### 4.4. UHPLC-HRMS/MS Analysis

Determination of PSP toxins in bivalve molluscs was performed by an UHPLC Dionex Ultimate 3000, equipped with a solvent reservoir, a quaternary pump, a refrigerated autosampler and a column oven, coupled with a Q-Exactive Orbitrap Plus high resolution mass spectrometer (Thermo Fisher Scientific, Waltham, MA, USA). The chromatographic separation of the 14 PSP toxins was performed by Hydrophilic Interaction Liquid Chromatography (HILIC). The chromatographic separation was carried out on a TSK gel Amide 80 column 2.0 × 150 mm, 5 µm particle size from Tosoh Corporation (Tokyo, Japan), heated at 34 °C, in gradient elution using the mobile phases water (A) and acetonitrile/water 95/5 *v*/*v* (B), both containing 3.6 mM formic acid and 0.2 mM ammonium formate. For analysis, 10 µL of sample were injected into the UHPLC system. The elution gradient is described in Table S3; during the analysis, at time 9.1 min, the flow rate varied from 0.5 to 0.7 mL min^−1^ to improve peak resolution.

Mass spectrometry analysis was carried out by electrospray ionization (ESI), both in positive and negative mode. The ion spray source parameters were set as shown in Table S4.

Optimization of the mass spectrometer parameters was attained by direct infusion of standard solutions containing the single PSP toxins studied. The standard solutions were in HCl or acetic acid at pH between 5 and 6; they were infused into the electrospray source at 10 μL min^−1^ flow rate using an external pump syringe. The mass spectra were obtained by scanning the *m*/*z* values in the range between 50.00 and 800.00 amu. In addition, standard solutions dissolved in the mobile phases were also infused to investigate their effects on ionization and fragmentation patterns of the analytes. The possible formation of adduct ions and their intensity compared to their respective precursor ions were also investigated. Moreover, experiments in both positive and negative ionization mode were performed to select the more intense product ions. The following ion spray source parameters were set for both positive and negative ion mode: Capillary Temperature at 350 °C; Sheath Gas rate at 50 psi; Auxiliary Gas rate at 30 psi; Auxiliary gas Heather Temperature at 350 °C; Ion Spray Voltage at ± 4.0 kV. During the analysis, the mass spectrometer worked at a resolution of 70,000 (*m*/*z* 200, FWHM). The toxins GTX 1/4, GTX 2/3, GTX 5, GTX 6, C 1/2 e dc-GTX 2/3 were determined in negative ion mode, STX, dc-STX, NEO, dc-NEO in positive ion mode. For each toxin, the precursor ion and two products ions were selected and monitored; in this study, all data were acquired in a combined full scan MS and target SIM data-dependent MS/MS (tSIM-ddMS2) scan mode, for both identification and quantification. The UHPLC-HRMS system was manged by the Xcalibur software version 4.0.27.19 (Thermo Fisher Scientific). In this mode, the quadrupole before the Orbitrap acquires SIM scans based on the selected accurate mass inclusion list. The first SIM scan is without fragmentation in the HCD, the second scan is a data-depend (dd) scan and ions enter into the HCD collision cell for the fragmentation. The data of the mass spectrometry optimization are reported in Table 1. For quantification, the precursor ion of each toxin was monitored, and the concentrations in samples were calculated by interpolation of the linear regression of matrix-matched calibration curves; the measurement performed on the basis of accurate mass, only if confirmed by the presence of corresponding MS/MS diagnostic product ions, allows for highly selective and sensitive determination.

### 4.5. Method Validation

The method described was validated according to the requirements of the Regulation (EU) n. 2017/625, Annex III [21], regarding the analytical performance for the official control of food, and to the guideline EURACHEM “The Fitness for Purpose of Analytical Methods” [22]. In particular, method selectivity, trueness in terms of mean recoveries, repeatability and within-laboratory reproducibility, expressed as relative standard deviations RSD_r_ and RSD_R_, respectively, linearity of detector response, and the limits of quantification (LOQs) were evaluated and calculated. The method was validated in-house testing sample and reagent blanks, as well as mollusc samples spiked at two different contamination levels. To assess selectivity, 20 samples of different bivalve molluscs (mussels, clams, razor clams, oysters) available in our laboratories and previously resulted not contaminated by PSP toxins were selected to assess selectivity.

The linearity of detector response was tested analysing several standard calibration curves, both in solvent and matrix-matched. For each PSP toxin, six-point calibration curves were calculated and compared. All the standards were injected in duplicate. The concentration range for each analyte was reported in Table 2.

The LOQs for all the PSP toxins were evaluated referring to the values reported in the reference Lawrence method AOAC n. 2005.06 [11] and the standard UNI EN 14526: 2017 [23]. These LOQs were experimentally verified and confirmed by our method, by analysing blank mollusc samples spiked at the same concentrations (3 replicates).

Method trueness and precision were calculated analysing blank samples from different uncontaminated molluscs, spiked with the 14 PSP toxins added before the extraction step. The spiking was carried out at two different levels, that is at concentrations equal to the LOQs for the PSP toxins and at concentrations about 10-fold higher than the LOQs, as reported in detail in Table 3.

### 4.6. Determination of PSP Toxins

Quantitative analysis was performed by the matrix-matched external standard calibration curve method, reporting peak areas of each matrix-matched standard *vs* concentrations; the concentration of each PSP toxin (TC) in the samples was calculated by interpolation of the linear regression calibration curve using the software Xcalibur version 4.0.27.19 (Thermo Fisher Scientific). Moreover, for each toxin other than STX, the saxitoxin equivalent concentration (STX_eq_) can be calculated correcting by the respective TEF proposed by the EFSA ([8]; Table S4), applying the following equation:

µg STX_eq_ kg^−1^ = TC (µg kg^−1^) × 1000/MW_toxin_ (g mol^−1^) × TEF/372.2 (g mol^−1^)

where TC is the toxin concentration, MW is the molecular weight of the toxin, 372.2 g mol^−1^ is the molecular weight of saxitoxin. For assessing sample compliance to the maximum tolerable limit set by the European Union, the total saxitoxin equivalent concentration must be calculated.

### 4.7. Sampling

**Figure 4:**
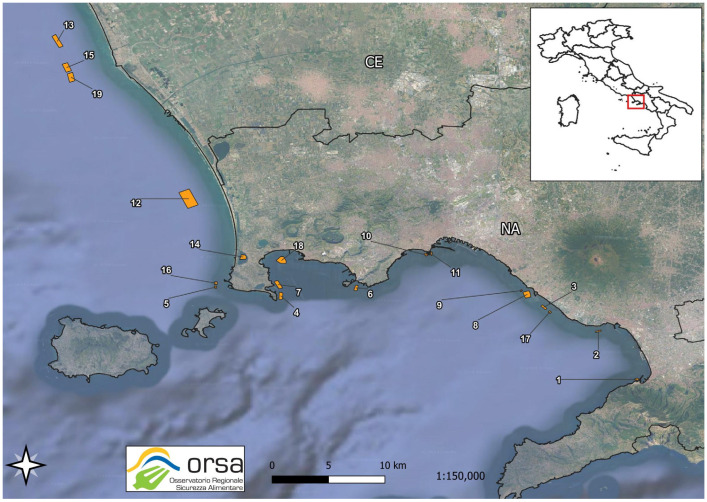
The location of the 19 mussel farms along the coasts of Campania Region (map from our Food Safety Regional Observatory—ORSA data bank); they are numbered according to the identification numbers in our Observatory.

The method after validation was accredited according to the UNI EN ISO/IEC 17025:2018 standard and used for the official control of mussels (*Mytilus galloprovincialis*) breed in Campania Region, where they represent an important economic resource. Between 2022 and the first six months of 2024, we tested 487 samples of *Mytilus galloprovincialis*, collected by the Local Health Authorities from 19 mussel farms along the coastal areas of the Region (Figure 4), for the planned official control activities. The samples were collected evenly during all the months of the year, because no algal bloom outbreak was observed. The farms are located in the areas of two provinces, that is Naples (NA) and Caserta (CE).

## Figures and Tables

**Table 1:** Mass spectrometry parameters for the targeted HRMS/MS analysis of 14 PSP toxins.

PSP Toxin	Ionisation Polarity	Precursor Ion (*m*/*z*)	Product Ions (*m*/*z*)	NCE ^1^ (%)
STX	+	300.14148	204.088–179.092	50
dc-STX	+	257.13556	222.098–180.077	52
NEO	+	316.13639	225.109–298.145	46
dc-NEO	+	273.13058	262.959–244948	40
GTX 1/4	-	410.07865	367.067–349.056	24
GTX 2/3	-	394.07865	333.062–351.072	25
GTX 5	-	378.08374	360.072–378.083	31
GTX 6	-	394.07865	121.954–150.055	25
C 1/2	-	474.03547	351.072–394.078	34
dc-GTX 2/3	-	351.07284	333.062–164.045	37

^1^ Normalised Collision Energy.

**Table 2:** The parameters of the linear regression standard matrix-matched calibration curves for the 14 PSP toxins.

PSP Toxin	Retention Time (min)	Standard Concentrations (µg mL^−1^)	Calibration Curve	r^2^
STX	10.71	4.1–164	Y = −140080 + 567550X	0.9995
dc-STX	10.70	3.6–144	Y = −552944 + 837888X	0.9980
GTX 1	4.80	3.9–156	Y = 160732 + 163409X	0.9983
GTX 2	4.60	3.4–136	Y = 257098 + 228682X	0.9980
GTX 3	5.32	1.4–56	Y = 162432 + 188204X	0.9966
GTX 4	5.56	1.2–48	Y = −18379.7 + 195033X	0.9999
GTX 5	6.51	3.5–140	Y = 18707.2 + 112114X	0.9986
GTX 6	7.28	3.5–140	Y = −19709.4 + 77072X	0.9991
NEO	10.74	3.3–132	Y = 104803 + 180342X	0.9991
dc-NEO	10.72	3.4–136	Y = −123596 + 68410.6X	0.9993
dc-GTX 2	4.97	2.9–116	Y = 268316 + 300984X	0.9945
dc-GTX 3	5.67	0.9–36	Y = −103641 + 313741X	0.9985
C 1/2 (sum)	1.63	4.7–188	Y = −1667.29 + 8727.4X	0.9982

**Table 3:** Results of the in-house validation study for the determination of 14 PSP toxins in bivalve molluscs.

PSP Toxin	Spiking Level (µg kg^−1^)	N. of Replicates	Mean Concentration (µg kg^−1^)	Mean Recovery (%)	RSDr (%)	RSD_R_ (%)
STX	21	6	19.5	92.9	2.8	-
	197	7	189.2	96.0	7.6	7.0
dc-STX	9	6	9.8	109.3	11.9	-
	86	7	96.2	111.9	4.8	4.8
GTX 1	32	6	29.8	93.2	5.8	-
	329	7	321.6	97.8	3.7	12.2
GTX 2	84	6	83.5	99.4	4.3	-
	811	7	806.2	99.4	3.0	6.0
GTX 3	36	6	36.2	100.5	5.4	-
	344	7	267.6	77.8	3.8	6.4
GTX 4	10	6	10.3	103.3	5.0	-
	104	7	88.2	84.8	2.2	10.2
GTX 5	29	6	28.0	96.6	3.9	-
	296	7	258.5	87.3	5.2	6.6
GTX 6	30	6	29.0	96.7	3.1	-
	313	7	262.9	84	7.0	8.0
NEO	43	6	38.50	89.5	2.2	-
	400	7	373.9	93.5	3.1	6.0
dc-NEO	44	-	-	-	-	-
	426	7	387	91	3.1	3.6
dc-GTX 2	29	6	27.2	93.7	2.8	-
	282	7	298.8	106.0	3.4	10.8
dc-GTX 3	9	6	8.7	96.3	6.0	-
	83	7	78.8	94.9	3.3	8.4
C1/2	97	6	89.3	92.1	8.5	-
	980	7	875.1	89.3	2.9	5.5

**Table 4:** The comparison of results from the proficiency test by NRL-BM carried out using the UHPLC-HRMS/MS method and the HPLC-FLD Lawrence reference AOAC n. 2005.06 method.

UHPLC-HRMS/MS Method				
PSP Toxin	CRM/01 (µg kg^−1^)	CRM/02 (µg kg^−1^)	CRM/03 (µg kg^−1^)	Recovery (%)
STX	23 ± 10	<LOQ	<LOQ	110
GTX 1	<LOQ	208 ± 84	<LOQ	80
GTX 4	<LOQ	69 ± 30	<LOQ	104
GTX 1/4 (sum)	<LOQ	274 ± 107	<LOQ	84
GTX 2	318 ± 121	248 ± 98	<LOQ	96
GTX 3	116 ± 51	91 ± 40	<LOQ	90
GTX 2/3 (sum)	433 ± 157	337 ± 127	<LOQ	94
GTX 5	439 ± 159	<LOQ	<LOQ	87
C 1/2 (sum)	1203 ± 374	<LOQ	<LOQ	99
STX equivalents^1^	363 ± 67	431 ± 156	<LOQ	-
**HPLC-FLD Lawrence Reference Method**				
**PSP Toxin**	**CRM/01** **(µg kg^−1^)**	**CRM/02** **(µg kg^−1^)**	**CRM/03** **(µg kg^−1^)**	**Recovery (%)**
STX	25 ± 11	<LOQ	<LOQ	92.2
GTX 1/4 (sum)	<LOQ	544 ± 148	<LOQ	92.2
GTX 2/3 (sum)	345 ± 130	441± 133	<LOQ	85.7
GTX 5	402 ± 148	<LOQ	<LOQ	96.9
C 1/2 (sum)	1078 ± 341	<LOQ	<LOQ	80.5
STX equivalents	400 ± 94	635 ± 217	<LOQ	-

The total concentrations are expressed as µg STXeq kg^−1^, corrected for recovery.

## Data Availability

The data presented in this study are available on request from the corresponding author. (The data are not publicly available due to privacy).

## Supplementary Materials

The following supporting information can be downloaded at: https://www.mdpi.com/article/10.3390/toxins16110502/s1, Table S1: Stock standard aqueous solutions of the PSP toxins studied; Table S2: Working standard solutions containing the PSP toxins studied; Table S3: UHPLC gradient elution program for PSP toxins analysis; Table S4: Toxicity equivalent factors (TEFs) proposed by the EFSA Panel, to be applied on a molar basis.
