# Supplementary material for: Paralytic Shellfish Poisoning (PSP) Toxins in Bivalve Molluscs from Southern Italy Analysed by Liquid Chromatography Coupled with High-Resolution Mass Spectrometry (UHPLC-HRMS/MS)
Source: Toxins (Basel). 2024 Nov 20;16(11):502. doi: 10.3390/toxins16110502 (PMC11598748; doi:10.3390/toxins16110502)
Supplement: Supplementary file 1 [file toxins-16-00502-s001.zip › toxins-3274393-supplementary.pdf]

Supplementary materials

Paralytic Shellfish Poisoning (PSP) Toxins in Bivalve Molluscs from Southern Italy Analysed by Liquid Chromatography Coupled with High-Resolution Mass Spectrometry (UHPLC-HRMS/MS)

Pasquale Gallo, Sara Lambiase, Ida Duro, Mauro Esposito and Angela Pepe

Table S1: Stock standard aqueous solutions of the PSP toxins studied.

PSP Toxin	Certified concentration (μg mL^{-1})	Volume (μL)	Solvent (μL)	Total Volume (μL)	Final Concentration (μg mL^{-1})
**STX**	9.84	125	305	1000	1.23
**dc-STX**	21.39	50			1.07
**GTX 1**	23.51	50			1.18
**GTX 4**	7.40				0.37
**GTX 1 / 4 (sum)**	30.91				1.55
**GTX 2**	40.53	25			1.01
**GTX 3**	17.18				0.43
**GTX 2 / 3 (sum)**	57.71				1.44
**GTX 5**	21.11	50			1.06
**GTX 6**	5.21	200			1.04
**NEO**	20.00	50			1.00
**dc-NEO**	10.14	100			1.01
**dc-GTX 2**	35.24	25			0.88
**dc-GTX 3**	10.35				0.26
**dc-GTX 2 / 3 (sum)**	45.58				1.14
**C 1**	53.87	20			1.08
**C 2**	16.10				0.32
**C 1 / 2 (sum)**	69.97				1.40

Table S2: Working standard solutions containing the PSP toxins studied.

	Working standard solutions					
	**1**	**2**	**3**	**4**	**5**	**6**
**Mobile phase B** (µL)	1200	1200	1200	1200	1200	1200
**Stock standard solution** (µL)	5	10	20	50	100	200
**Blank matrix solution** (µL)	295	290	280	250	200	100
**GTX 1** (µg mL^{-1})	0.0039	0.0078	0.0156	0.039	0.078	0.0156
**GTX 4** (µg mL^{-1})	0.0012	0.0024	0.0048	0.012	0.024	0.048
**NEO** (µg mL^{-1})	0.0033	0.0066	0.0132	0.033	0.066	0.132
**GTX 2** (µg mL^{-1})	0.0034	0.0068	0.0136	0.034	0.068	0.136
**GTX 3** (µg mL^{-1})	0.0014	0.0028	0.0056	0.014	0.028	0.056
**STX** (µg mL^{-1})	0.0041	0.0082	0.0164	0.041	0.082	0.164
**dc-STX** (µg mL^{-1})	0.0036	0.0072	0.0144	0.036	0.072	0.144
**GTX 5** (µg mL^{-1})	0.0035	0.0070	0.0140	0.035	0.070	0.140
**dc-GTX 2** (µg mL^{-1})	0.0029	0.0058	0.0116	0.029	0.058	0.116
**dc-GTX 3** (µg mL^{-1})	0.0009	0.0018	0.0036	0.009	0.0018	0.0036
**dc-NEO** (µg mL^{-1})	0.0034	0.0068	0.0136	0.034	0.068	0.136
**C 1 / 2** (µg mL^{-1})	0.0047	0.0094	0.0188	0.047	0.094	0.188
**GTX 6** (µg mL^{-1})	0.0035	0.0070	0.0140	0.035	0.070	0.140

Table S3: UHPLC gradient elution program for PSP toxins analysis.

Time (min)	Flow rate (mL min^{-1})	Mobile phase A (%)	Mobile phase B (%)
0.0	0.5	36	64
8.0	0.5	36	64
8.1	0.5	30	70
9.0	0.5	30	70
9.1	0.7	80	20
19.0	0.7	80	20
19.5	0.5	36	64
25.0	0.5	36	64

Mobile phase A: water containing 3.6 mM formic acid and 0.2 mM ammonium formate.

Mobile phase B: Acetonitrile-Water (95/5 v/v) containing 3.6 mM formic acid and 0.2 mM ammonium formate.

Table S4: Toxicity equivalent factors (TEFs) proposed by the EFSA Panel, to be applied on a molar basis.

Toxin	TEF	Molecular weight (g mol^{-1})
**STX**	1.0	372.2
**NEO**	1.0	315.1
**GTX 1**	1.0	411.4
**GTX 2**	0.4	395.4
**GTX 3**	0.6	395.4
**GTX 4**	0.7	411.4
**GTX 5**	0.1	379.35
**GTX 6**	0.1	395.35
**dc-STX**	1.0	329.2
**dc-NEO**	0.4	345.2
**dc-GTX 2**	0.2	352.3
**dc-GTX 3**	0.4	352.3
**C1 / 2**	0.1	476.05
